# Diagnosis of Pulmonary Embolism during Pregnancy

**DOI:** 10.3390/diagnostics12081875

**Published:** 2022-08-03

**Authors:** Helia Robert-Ebadi, Thomas Moumneh, Grégoire Le Gal, Marc Righini

**Affiliations:** 1 Division of Angiology and Hemostasis, Department of Medicine, Geneva University Hospitals and Faculty of Medicine, 4, Rue Gabrielle-Perret-Gentil, CH-1211 Geneva, Switzerland; marc.righini@hcuge.ch; 2 Département de Médecine d’Urgence, CHU d’Angers, Institut MITOVASC, UMR CNRS 6015 UMR INSERM 1083, Université d’Angers, 49035 Angers, France; thomas.moumneh@chu-angers.fr; 3 Department of Medicine, Ottawa Hospital Research Institute, University of Ottawa, Ottawa, ON K1Y 4E9, Canada; glegal@toh.ca

**Keywords:** pulmonary embolism, diagnostic strategy, D-dimer, clinical probability, pregnancy, computed tomography pulmonary angiography, ventilation-perfusion lung scan

## Abstract

Although rare, pulmonary embolism (PE) remains one of the most common causes of severe maternal morbidity and mortality during pregnancy. Among pregnant women with suspected PE, the prevalence of confirmed disease is far lower than in the general population, reflecting the fear of missing the diagnosis and a low threshold to suspect PE in this setting. Two prospective management outcome trials have recently assessed two different diagnostic algorithms based on the assessment of clinical probability, D-dimer, venous compression ultrasonography of the lower limbs (CUS), and computed tomography pulmonary angiography (CTPA). Both demonstrated the safety of such strategies to exclude PE, with a very low failure rate defined as the rate of subsequent 3-month venous thromboembolism in women left untreated after a negative work-up. These studies were also the first to prospectively demonstrate the safety of negative D-dimer associated with a clinical prediction rule to exclude PE without any chest imaging. Pregnant women are known to be a subgroup at particularly high risk of inappropriate diagnostic management, so the implementation of such validated diagnostic strategies in clinical practice should represent a high priority goal.

## 1. Introduction

The diagnosis of pulmonary embolism during pregnancy is difficult as clinical presentation may be misleading and few prospective data is available. Hence, we performed a narrative review reporting recent knowledge in this challenging field. Women of childbearing age are at low risk of developing venous thromboembolism (VTE). Pregnancy however represents a period at risk, with an overall incidence of VTE estimated at 1/1000 pregnancies [1,2]. The risk is highest during the third trimester and the 6 to 12 weeks following delivery [3]. Many of the symptoms and signs reported in almost 50% of women during physiological pregnancy, such as shortness of breath or lower limb oedema—especially as pregnancy advances through the third trimester—may suggest the possibility of a pulmonary embolism (PE) and/or deep vein thrombosis (DVT) [4,5]. Syncope and pre-syncope are also reported by pregnant women [6]. Which clinical picture represents a clinical suspicion of PE is therefore even more difficult to define in pregnant women than in the general population. Most often, an acute onset of progressive dyspnea and/or chest pain without any other obvious explanation raises a clinical suspicion of PE, especially if additional transient risk factors of VTE (e.g., reduced mobility) have been present during the previous weeks.

### Epidemiological Data

Although VTE risk is increased 7 to 10-fold during pregnancy compared to age-matched controls, the absolute incidence remains low at around 1/1000 [7]. However, PE is responsible for around 10% of pregnancy-associated mortality. Nevertheless, the fear of missing a PE during pregnancy leads to a low threshold to suspect the disease. This is well reflected by the very low prevalence of confirmed disease among pregnant women with suspected PE in clinical trials, of around 2 to 7% [4,8,9], compared to a prevalence of 10 to 20% in diagnostic studies in the general population [10,11]. Interestingly, among pregnant women with suspected DVT, the prevalence of confirmed disease was also shown to be low, at 9 to 10% [5,12]. Another important point is that falsely diagnosing PE might be associated with bleeding complications that can be lethal for the mother and her baby. Furthermore, falsely diagnosing a PE in a young woman will have potential major implications regarding antithrombotic prophylaxis in case of further pregnancy or regarding future administration of contraceptive pills or hormone replacement therapy.

As more than 90% of pregnant women with suspected PE do not have PE, the central aim of diagnostic management of these patients lies in the *exclusion* of the diagnosis. Moreover, keeping in mind the low prevalence of confirmed disease, the least invasive and fewest radiating tests allowing a safe exclusion of PE should be applied. Of note, the reference standard currently used in clinical trials and recommended by the International Society on Thrombosis and Hemostasis (ISTH) to assess the safety of diagnostic strategies in VTE, is the rate of VTE events occurring during the 3 months following VTE exclusion, also called the “failure rate” of the strategy [13].

## 2. Diagnostic Management of Pregnant Women with Suspected Pulmonary Embolism

Until recently, all pregnant women with suspected PE historically underwent a thoracic imaging test [14]. The first “non-invasive” alternative to pulmonary angiography assessed in the 1990s was ventilation-perfusion lung scintigraphy (V/Q scan), in the hallmark PIOPED trial in a general population of patients with suspected PE [15]. Despite the absence of any scientific validation in pregnant women, perfusion only lung scintigraphy was adopted in clinical practice, based on the hypothesis that ventilation phases were highly likely to be normal in this young population with very low prevalence of cardio-pulmonary comorbidities. In a study published in 2006 assessing the appropriateness of diagnostic management of patients with suspected PE, pregnancy was by far the strongest predictor of inappropriate management, 69% of pregnant women with suspected PE being inappropriately managed [16]. This observation is most probably related to the lack of data and prospective studies performed specifically in this population [16]. More recently, two prospective management outcome studies published in 2018 [8] and 2019 [9] (see below) have started to fill in the knowledge gap in this setting. Indeed, these two studies represent the first prospective scientific validation of PE diagnostic algorithms in pregnant women with suspected PE.

### 2.1. Assessing the Pre-Test Clinical Probability of PE in Pregnant Women

Modern PE diagnostic algorithms are based on the assessment of pre-test clinical probability (PTP) as the first step in front of a patient with clinically suspected PE, and this crucial step is strongly supported by international guidelines [17,18]. The aim of these PTP prediction rules is to identify a group of patients with a low prevalence of PE in whom a negative D-dimer provides a high negative predictive value and safely excludes PE without imaging. Currently used PTP scores were derived and validated in PE diagnostic studies in which pregnancy represented an exclusion criterion [19]. The absence of scores validated in pregnant women is, as a matter of fact, one of the reasons refraining physicians from using D-dimer in this population.

**Figure 1:**
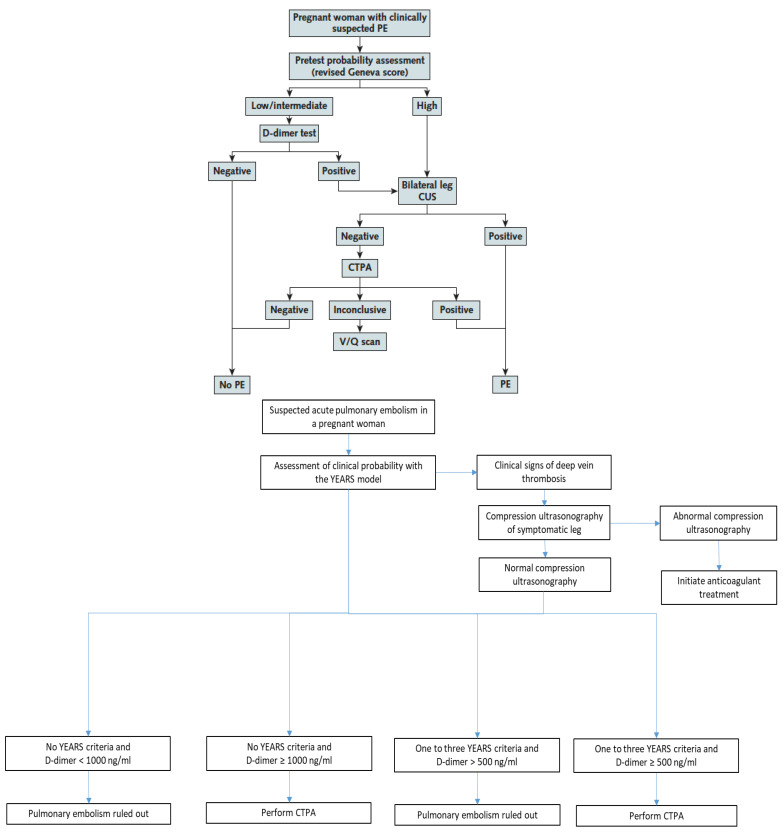
The CT-PE pregnancy and the pregnancy-adapted YEARS diagnostic algorithms [8,9].

As stated above, the CT-PE pregnancy and ARTEMIS studies were the first studies to prospectively assess diagnostic strategies in pregnant women with suspected PE [8,9]. They applied two different PTP assessment models—the Geneva score and a pregnancy-adapted YEARS model—that had not been previously derived nor validated in pregnant women. Nevertheless, these two clinical decision rules (CDRs) both proved their usefulness in the integrated diagnostic algorithms used in these two studies (Figure 1) [8,9].

External validation of the ARTEMIS model in the CT-PE pregnancy population confirmed the safety of this pregnancy-adapted YEARS model in a second cohort of patients [20]. In the CT-PE pregnancy study, all items of the Wells score had been prospectively collected, including the subjective item “PE is the most likely diagnosis” included in the three YEARS criteria. Interestingly, physicians in charge of patients had assessed PE as the most likely diagnosis in 71% of pregnant women with suspected PE in the CT-PE cohort, compared with only 44% in the ARTEMIS cohort [20]. This may be partly explained by a different assessment of the item depending on its influence or not on subsequent diagnostic management, and on the fact that D-dimer results were already known to the physician for some ARTEMIS study patients (knowledge of negative D-dimer results may have influenced the assessment of this item).

In the continuing endeavor to progressively develop CDRs for pregnant women with suspected PE, a novel PTP score—the Pregnancy-Adapted Geneva score (PAG score)—was recently proposed. The PAG score has the advantage of containing only *objective* items that are all relevant to pregnant women (see Table 1) [21]. It showed a good discriminatory power to classify pregnant women in three categories of PTP (low, intermediate, and high) corresponding to increasing prevalence of the disease (Table 1), and an area under the ROC curve of 0.80 (95% CI 0.690–0.899). However, the PAG score needs prospective validation before it can be used in clinical practice.

### 2.2. Safety and Usefulness of D-Dimer to Exclude PE during Pregnancy

Physicians tend to consider D-dimer tests as useless in pregnant women with suspected VTE. The lack of a PTP score validated in this population is one of the reasons why, as discussed above. However, the most frequent reason reported by clinicians is their belief that D-dimer does not represent a useful test in pregnant women due to the perception of D-dimer levels being always elevated during pregnancy. Moreover, even the safety of excluding VTE by a negative D-dimer test during pregnancy has been challenged by some authors [22].

The safe exclusion of PE by a negative D-dimer test associated with a non-high/unlikely PTP has been widely accepted outside pregnancy, based on large scale clinical trials demonstrating a very low 3-month VTE rate, and has been implemented in clinical practice for many years [10,11,23,24]. Although decreased intrinsic fibrinolysis during pregnancy has been raised as a hypothetical mechanism leading to a potential decrease in D-dimer levels, there is no true rationale that supports a lower sensitivity of D-dimer for the diagnosis of VTE during pregnancy.

The CT-PE pregnancy and ARTEMIS studies both showed the safety of excluding PE during pregnancy by a negative D-dimer test in sequential diagnostic algorithms (Figure 1) with low failure rates. The number of women included in these studies remains limited compared to studies in the general population, and prospective trials are still needed to enrich these data. Nevertheless, integrating data from these two trials with previous data on D-dimer as a VTE exclusion test during pregnancy in a meta-analysis, the high negative predictive value of D-dimer was confirmed. The pooled sensitivity and negative predictive values were 99.5% (95% CI 95.0–100.0%) and 100% (95% CI 99.19–100.0%), respectively [25].

Regarding the diagnostic yield—also called efficiency—of a negative D-dimer test associated with a non-high PTP to safely rule out PE without any additional tests (CUS or chest imaging) was reported at 12% in the CT-PE pregnancy study. In other words, the number of pregnant women needed to test with D-dimer to exclude one PE was 8.3 [8]. In a population in whom radiation exposure is a central concern, this efficiency is clinically relevant, as avoiding radiating tests by a simple blood test in 1 out of 8 pregnant women with suspected PE is already highly appealing.

The ARTEMIS study used a variable D-dimer cutoff depending on the presence or absence of YEARS criteria (Figure 1). Using a higher cutoff of 1000 ng/mL in women with no YEARS criteria, and a standard cutoff of 500 ng/mL in all other women, the efficiency of negative D-dimer to exclude PE without thoracic imaging was 39%. Of note, the non-invasive strategy tested in the ARTEMIS algorithm was not only based on PTP assessment and D-dimer measurement, but included the pre-exclusion of DVT by lower limb CUS in all women with clinical signs of DVT at baseline, representing a more complex selection of “low-risk” women in whom a higher D-dimer cutoff could be used to exclude PE (Figure 1) [9].

Pooling all the available evidence to assess efficiency of D-dimer to exclude VTE during pregnancy, the meta-analysis referred to in the last paragraph reported an overall efficiency of 34% (95% CI 15.9–55.23%) [25]. Although D-dimer levels are known to increase during physiological pregnancy as a result of a gradually increasing hypercoagulable state as pregnancy progresses towards delivery [7,26,27], they remain below the diagnostic cutoff for exclusion of VTE in a significant proportion of pregnant women, especially during the first half of pregnancy [26,27]. Giving a pregnant woman with a clinical suspicion of PE the chance to avoid a radiating thoracic imaging test should, in our opinion, not be neglected, and in spite of the controversies in international guidelines [28], we believe that the use of D-dimer measurements in the decision algorithm should be strongly encouraged.

### 2.3. The Diagnostic Yield of Lower Limb Compression Ultrasound in Pregnant Women with Suspected PE

Bilateral lower limb compression ultrasound (CUS) was part of the diagnostic strategies of both CT-PE pregnancy and ARTEMIS studies but integrated in a different manner (Figure 1).

In the CT-PE pregnancy study, CUS was performed systematically in all women with a high PTP, or a low-intermediate PTP associated with positive D-dimer, as the first-line “imaging” modality. If a proximal DVT was confirmed, PE diagnosis was considered as confirmed without chest imaging. If no proximal DVT was present, CTPA was performed. Overall, CUS was performed in 88% of the overall population. Interestingly, proximal DVT was found in only 2% of these unselected women. Among women with clinical symptoms and signs of DVT, proximal DVT was found in 9% [8].

In the ARTEMIS study, CUS was part of the algorithm at baseline in all women with clinical signs of DVT, representing 9% of the whole cohort of pregnant women with suspected PE. When performed in this setting, CUS identified proximal DVT in 7% of women with leg symptoms. Of note, CUS was also performed in an additional 16% of the whole cohort in women without leg symptoms (reason not known) and showed DVT in only 1% of this group [9].

These interesting observations confirm that CUS seems to be mainly useful in pregnant women with suspected PE *and* lower limb symptoms suggestive of DVT (pain +/− edema). Nevertheless, even a diagnostic yield of only 1–2% when performing systematic CUS in pregnant women with suspected PE that could not be excluded by PTP and D-dimer before moving on to chest imaging is considered worthwhile by some, if the focus is set on minimizing radiating tests than on cost-effectiveness [19].

Despite this limited utility, some but not all, international guidelines currently recommend bilateral CUS in pregnant women with suspected PE in whom PE diagnosis could not be excluded by the combination of PTP and D-dimer, and in whom further testing is thus needed before proceeding to chest imaging [17].

### 2.4. Thoracic Imaging in Pregnant Women with Suspected PE

Although the use of PTP scores and D-dimer reduces the need for chest imaging, a significant proportion of pregnant women with suspected VTE still require imaging [25]. The two imaging modalities validated in the setting of PE diagnosis outside pregnancy are CTPA and V/Q lung scintigraphy. CTPA has now become the new *gold standard* in the diagnostic management of PE in clinical practice [29] and the recommended imaging test in most international guidelines [25]. The wide accessibility of CTPA has even led to concerns about over-testing and consequent radiation exposure, as well as over-diagnosis of small PE, some of which are of uncertain clinical significance. In pregnant women with suspected PE, radiation exposure both to the mother and the fetus are the first matter of concern [30], the second being the rate of inconclusive results.

A meta-analysis compared all available data on CTPA versus V/Q lung scintigraphy in pregnant women. No conclusion could be drawn regarding relative radiation risks between CTPA and V/Q lung scan due to the wide heterogeneity in radiation dose calculation methods and imaging protocol used across studies [30]. Nevertheless, this work highlighted the fact that all reported radiation doses for CTPA and V/Q lung scan, including older studies using protocols with no adaptation for pregnant women, were far below the accepted harmful threshold of 100 mGy [30]. The proportion of inconclusive tests varied very widely across the individual studies included in this meta-analysis both for CTPA (0–57%) and V/Q scan (1–40%). The pooled proportion of non-diagnostic results were similar between CTPA (12%, 95%CI 6–17%) and V/Q scan (14%, 95% CI 10–18%) [30]. Beyond the debate on the optimal imaging modality to use during pregnancy, experts and scientific societies all agree that the risks of an inappropriate diagnostic work-up, leading either to a missed diagnosis or to the introduction of therapeutic anticoagulation based only on clinical grounds, by far outweigh the risk of any of these two diagnostic modalities [14,31].

Over the last two decades, CTPA technology has considerably evolved, leading to dramatically lower radiation exposure than the first generation CTPAs. Adapted protocols have also been developed for pregnant women, including reduced kilovoltage and reduced anatomical coverage of the scanned area. In order to reduce non-diagnostic tests related to hemodynamic changes during pregnancy, the most recent protocols typically use high-concentration contrast media, with high-volume and high-rate of injection immediately followed by saline flush. Images are acquired instructing the patient to breath-hold during shallow inspiration to avoid the hemodynamic consequences of a Valsalva maneuver on venous return and pulmonary tree opacification [32]. Interestingly, the reported risk of inconclusive CTPA was 7% in the CT-PE pregnancy study and 0% in the ARTEMIS study, so far below the numbers reported in the meta-analysis cited above. Nuclear medicine techniques have also evolved considerably since their introduction in clinical practice in the early 1990s. Single-photon emission computed tomography (SPECT) is currently under investigation outside pregnancy in the SPECTACULAR study (NCT02983760), which is the first prospective management outcome study to assess SPECT in a diagnostic algorithm for suspected PE, and compares its diagnostic performance to CTPA and the classical planar V/Q lung scintigraphy with a randomized design.

Despite the ongoing optimization of CTPA protocols for suspected PE during pregnancy, the historical belief of significantly higher radiation doses to the mother’s breast tissue remains strongly embedded in physician’s minds and still influences the choice towards V/Q lung scintigraphy in some geographic areas, such as the United States. The choice for V/Q lung scintigraphy as the first imaging modality is also strongly driven by local expertise and availability of the nuclear medicine team. Given the very low prevalence of cardio-pulmonary comorbidities in young pregnant women, a two-step protocol is used in some centers: a perfusion only scan is performed, and PE considered as excluded if the perfusion scan is normal (especially if chest X-Ray is performed and is also normal); ventilation sequences are only performed if the perfusion pattern is abnormal to confirm or exclude a mismatch suggestive of PE. It should however be emphasized that this stepwise strategy has never been assessed in prospective studies and caution is required. This particularly applies in case of an abnormal perfusion pattern: indeed, PE diagnosis should not be considered as confirmed without formal exclusion of any ventilation abnormality or parenchymal abnormality in the same area that would be highly suggestive of an alternative diagnosis. In case of an inconclusive CTPA, a perfusion lung scan is often proposed as a second test in recent studies which validated CTPA in this setting.

Overall, CTPA is now better validated than V/Q scan or perfusion scan alone in the diagnosis of PE in pregnant women. However, in some centers with valuable expertise and availability of nuclear medicine, V/Q scan remains an interesting alternative.

### 2.5. Scientific Societies’ Recommendations for PE Diagnosis during Pregnancy

The 2018 American society of Hematology (ASH) guidelines for PE diagnosis were strongly driven by the willingness to minimize radiation even in the general population, and thus advocate for V/Q scans for patients likely to have a diagnostic scan and in centers where V/Q scans are available with expertise to interpret the results in a timely manner [18].

The 2019 European Society of Cardiology (ESC) Guidelines provide specific recommendations for pregnant women [17]:

- formal diagnostic assessment with validated methods (Class I, level B)
- D-dimer measurement and clinical prediction rules to rule out PE (Class IIa, level B)
- venous CUS to avoid unnecessary irradiation (Class IIa, level B)
- and in terms of imaging test: perfusion scintigraphy or CTPA (with a low-radiation dose protocol); CTPA as the first-line option if chest X-ray is abnormal (Class IIa, level C) recommendation.

## 3. Recently Validated Diagnostic Algorithms in Pregnant Women with Suspected PE

Only two prospective management outcome studies have been published to date in pregnant women with suspected PE: the CT-PE pregnancy and ARTEMIS studies referred to throughout this manuscript [8,9]. Their diagnostic algorithms are presented below and in Figure 1.

### 3.1. The CT-PE Pregnancy Study

The CT-PE pregnancy study was published in 2018 [8] and included 395 pregnant women with clinically suspected PE. The diagnostic algorithm applied a sequential assessment of clinical PTP using the revised Geneva score and a high-sensitivity D-dimer test with a standard cutoff of 500 ng/mL. PE was considered excluded in patients with a non-high PTP and negative D-dimer. In patients with a high PTP or low/intermediate PTP and a positive D-dimer test, lower limb venous CUS was performed regardless of the presence of leg symptoms or signs. If a proximal DVT was shown, PE was considered as confirmed without further testing. In the absence of proximal DVT, CTPA was performed (Figure 1). PE prevalence was 7.1%, and the failure rate of the strategy was 0.0% (95% CI 0.0–1.0%). The overall percentage of pregnant women with suspected PE managed without thoracic imaging was 14%.

### 3.2. The ARTEMIS Study

The ARTEMIS study was published in 2019 [9] and included 498 women with clinically suspected PE. A pregnancy-adapted YEARS model was applied (Figure 1) starting with D-dimer measurement and assessment of the presence of any of the 3 YEARS items (clinical signs of DVT, hemoptysis, PE as the most likely diagnosis). Patients with clinical signs of DVT all underwent CUS already at this stage. A higher D-dimer cutoff of 1000 ng/mL was used in women with no YEARS items and the standard cutoff of 500 ng/mL in all other women. PE prevalence was 4%, and the failure rate of the strategy was 0.21% (95% CI 0.04–1.2%). The percentage of patients with negative D-dimer according to the algorithm was 39%.

A detailed discussion on the strengths and limitations of these two studies has been previously reported [19] and some important observations highlighted throughout different sections of this review. The most important strength of these studies is to be the first prospective studies to assess standardized diagnostic algorithms in pregnant women with suspected PE and fill in this knowledge gap in this population. Extending prospective data in pregnant women is likely to contribute to a better appropriateness of their diagnostic management in the future.

Of note, external validation of the ARTEMIS model in the CT-PE pregnancy cohort showed that none of the women with a negative YEARS algorithm had PE either at initial testing or during the 3-month follow-up. The efficiency of D-dimer was, however, not as high as reported in the original study (21% in the CT-PE pregnancy cohort as compared with 39% in the ARTEMIS study) [20].

## 4. Remaining Controversies and Future Perspectives

The optimal diagnostic strategy in presence of a clinically suspected PE during pregnancy remains a highly debated topic. Until recently, guidelines remained controversial on the use of D-dimer to exclude VTE during pregnancy. The CT-PE pregnancy and ARTEMIS models have also been challenged in an analysis performed on a UK cohort of 219 patients including pregnant women with PE diagnosed primarily by imaging, with the conclusion that none of the two models was safe to exclude PE during pregnancy. To provide some context for the readers who may not be familiar with these different cohorts, the original DiPEP study this retrospective analysis was performed on had several limitations. Most importantly, the DiPEP cohort was not a purely prospective cohort, different D-dimer tests with variable cutoffs were used, and there was no standardized diagnostic algorithm [33]. As part of the cohort was represented by pregnant women with confirmed PE, the prevalence is high (41%) in this cohort and does not reflect the prevalence of PE among pregnant women presenting with suspected PE, limiting the interpretation of negative predictive values [13] and their comparison with prospective trials. The reported messages from the recent analysis performed on the DiPEP cohort can therefore not truly challenge the results of the prospective management outcome trials, as would be the case if a true external validation cohort had been used, and the conclusion against D-dimer use based on this data should be interpreted with caution.

Persistent concern about the use of diagnostic tests associated with maternal and fetal radiation exposure leads to ongoing debates and controversies. While fetal exposure seems to be in the same range with both tests, CTPA is more radiating for the mother’s breasts. No additional risk of early-onset breast cancer has been observed in a large population cohort study with a median follow-up of almost 6 years after CTPA and of 7.3 years after V/Q scan. These findings per se might however be considered insufficient due to the long but limited duration of follow-up, and the absence of information on the risk of repeated chest imaging in the same patient [34]. The level of scientific validation of CTPA is now higher and more extensive than V/Q scans. Moreover, logistic limitations come into consideration in clinical practice. Urgent access to a V/Q scan beyond day working hours is currently difficult in most centers, even in large teaching hospitals, whereas CTPA is easily available 24 h a day in most emergency centers. Finally, CTPA may reveal an alternative diagnosis in patients without PE. For all these reasons, CTPA is likely to become the most widely used diagnostic test in the majority of pregnant women with suspected PE, in whom the diagnosis could not be excluded by PTP and D-dimer. Ongoing progress and future perspectives in this field are represented by the gradually decreasing reported radiation doses to the maternal breast with modern CTPA techniques, and the assessment of the safety and usefulness of low-dose CTPA protocols in pregnant women (OPTICA study, NCT 04179487) [35].

## 5. Conclusions

In conclusion, important progress has been achieved during the last few years in the diagnostic management of pregnant women with suspected PE, with the publication of the first prospective management outcome studies in this population. These data need to be further expanded in additional prospective trials, with the aim of refining algorithms and maximizing the diagnostic yield of non-radiating strategies using clinical decision rules specifically validated in pregnant women, as well as D-dimer. In the meantime, a priority is to favor implementation of the already acquired knowledge into clinical practice and increase the appropriateness of management of pregnant women presenting with clinically suspected PE.

## Figures and Tables

**Table 1:** The Pregnancy-Adapted Geneva score for assessment of pre-test clinical probability of PE in pregnant women [21].

The Pregnancy-Adapted Geneva Score			
ITEM			POINTS
Age 40 years and older			+1
Surgery (under GA) or lower limb fracture in past month			+2
Previous DVT or PE			+3
Unilateral lower limb pain			+3
Hemoptysis			+2
Pain on lower limb palpation and unilateral oedema			+4
Heart rate > 110 bpm			+5
Maximal point number			20
**Points**	**PTP Category**	**PE Prevalence in Development Cohort**	**95% CI**
0–1	Low	2.3%	1.0–4.9%
2–6	Intermediate	11.6%	6.9–18.9%
≥7	High	61.5%	35.5–82.2%
